# Multifactorial Regulation of a Hox Target Gene

**DOI:** 10.1371/journal.pgen.1000412

**Published:** 2009-03-13

**Authors:** Petra Stöbe, Sokrates M. A. Stein, Anette Habring-Müller, Daniela Bezdan, Aurelia L. Fuchs, Stefanie D. Hueber, Haijia Wu, Ingrid Lohmann

**Affiliations:** 1 Department of Molecular Biology, Max Planck Institute for Developmental Biology, Tübingen, Germany; 2 BIOQUANT Center, Heidelberg, Germany; University of Pennsylvania School of Medicine, United States of America

## Abstract

Hox proteins play fundamental roles in controlling morphogenetic diversity along the anterior–posterior body axis of animals by regulating distinct sets of target genes. Within their rather broad expression domains, individual Hox proteins control cell diversification and pattern formation and consequently target gene expression in a highly localized manner, sometimes even only in a single cell. To achieve this high-regulatory specificity, it has been postulated that Hox proteins co-operate with other transcription factors to activate or repress their target genes in a highly context-specific manner *in vivo*. However, only a few of these factors have been identified. Here, we analyze the regulation of the cell death gene *reaper* (*rpr*) by the Hox protein Deformed (Dfd) and suggest that local activation of *rpr* expression in the anterior part of the maxillary segment is achieved through a combinatorial interaction of Dfd with at least eight functionally diverse transcriptional regulators on a minimal enhancer. It follows that context-dependent combinations of Hox proteins and other transcription factors on small, modular Hox response elements (HREs) could be responsible for the proper spatio-temporal expression of Hox targets. Thus, a large number of transcription factors are likely to be directly involved in Hox target gene regulation *in vivo*.

## Author Summary

Bilateral animals share a common genetic mechanism to control development along the anterior–posterior body axis, and transcription factors of the Hox class are key regulators of this conserved process. It is thought that Hox proteins drive morphological diversification of body segments by differentially controlling the expression of downstream genes. However, due to their highly conserved DNA binding domain, the homeodomain, Hox proteins alone bind to very similar and frequently occurring sequences in the genome. This implies that Hox proteins alone are likely insufficient to activate or repress their target genes in a proper spatio-temporal fashion. In contrast to this observation, Hox proteins have very specific effects during development and execute their function with high precision. A solution to this paradox could lie in the context-specific interaction of Hox proteins with other transcriptional regulators; however, only a few examples are known. By analysing the mechanism underlying the regulation of the Hox target gene *reaper*, we identified a set of eight transcription factors to be important for the precise spatio-temporal regulation of this gene. Based on our findings, we suggest that Hox proteins functionally interact with a plethora of unrelated transcription factors on small, yet complex enhancer elements to execute their specific functions throughout development of diverse organisms.

## Introduction

Distinct morphological structures exist along the anterior-posterior (A/P) axes of animals, and the *Hox* genes represent the major regulators for patterning of this body axis in organisms as diverse as fruit flies, fish and humans [1],[2],[3]. Since *Hox* genes code for transcription factors, Hox-dependent morphogenesis is driven by the differential regulation of downstream genes [4],[5],[6]. In line with the very diverse and many-fold effects of Hox proteins on morphogenesis, Hox transcription factors are known to regulate a large number of Hox downstream genes [7],[8], including genes that themselves have broad effects on morphology, as well as genes involved in terminal differentiation [reviewed in 3].

*Hox* genes are expressed in broad and partially overlapping domains along the A/P axis [1],[2],[3], and their constant and simultaneous activity within hundreds of cells is required throughout development [1]. Despite being active in a very large number of cells, Hox proteins affect target gene expression in precisely defined sub-domains in the animal [for example 9],[10],[11],[12],[13],[14],[15]. In the most extreme case the regulation of a Hox target gene can be limited to a single cell [16]. In addition, some downstream genes can be activated and repressed by the same Hox protein depending on the tissue or developmental stage. Finally, this context dependency also allows a single Hox protein to affect distinct sets of target genes in the same cells during the course of development [8],[11],[17]. While more and more of these complex regulatory interactions are being described, the molecular mechanisms underlying the spatio-temporal precision of Hox target gene regulation is only poorly understood. This is in large part due to our limited knowledge of the design and function of Hox-dependent enhancers and promoters and their interaction with the regulatory environment.

Only a few Hox regulated enhancers have been analyzed in some detail in *Drosophila* [3]. For example, the activation of *wingless* (*wg*) expression in the visceral mesoderm of *Drosophila* embryos had been shown to be dependent on the Hox protein Abdominal-A (Abd-A) and the Dpp/TGF-β signalling pathway, with both activities functioning on a small *wg* enhancer [11]. Here, two transcriptional effectors of the Dpp/TGF-β pathway, Mother against dpp (Mad) and Creb, had been shown to mediate the Dpp response on the *wg* enhancer, and were thus assumed to work in concert with the Hox protein Abd-A [11]. Only recently, Mad and another effector of the Dpp/TGF-β pathway, Medea (Med), have been found to collaborate with the Hox protein Ultrabithorax (Ubx) to repress transcription of the Hox target gene *spalt major* (*sal*) in the haltere by independently interacting with adjacent Mad/Med and Ubx binding sites in a small *sal* enhancer [18]. And finally, two transcription factors very well known for their function in the *Drosophila* segmentation cascade, Engrailed (En) and Sloppy paired 1 (Slp1), were shown to assist the Hox proteins Ubx and Abd-A in repressing *Distal-less* (*Dll*) expression in the abdomen of *Drosophila* embryos by occupying their identified binding sites in a minimal *Dll* enhancer [10].

Another well-studied direct Hox target gene in *Drosophila* is the apoptosis gene *rpr*, *which* is activated by the Hox protein Dfd in the anterior part of the maxillary segment through four binding sites located in the *rpr*-4S3 regulatory fragment [13]. Since Dfd is active throughout the maxillary segment, whereas *rpr* RNA is found only locally [19], it seemed likely that additional factors contribute to region specific Dfd-dependent *rpr* expression. Here, we find that eight transcriptional regulators, with diverse roles in patterning or differentiation processes, co-operate with Dfd in the regulation of *rpr*. Within their spatially restricted expression domains, these regulators are recruited to a minimal *rpr* enhancer through specific cis-regulatory DNA sequences and act together with Dfd to regulate *rpr* expression in the appropriate spatio-temporal pattern. Thus, our data support the idea that the combinatorial activity of Hox proteins and diverse transcriptional regulators on small regulatory elements is responsible for the spatially and temporarily restricted expression of Hox target genes *in vivo*. In addition, our data show that even small Hox-dependent enhancers are complex and integrate diverse regulatory inputs, which result in precise spatio-temporal expression of Hox target genes.

## Results

### Dissection of the Dfd-Dependent *rpr* Enhancer

**Figure 1:**
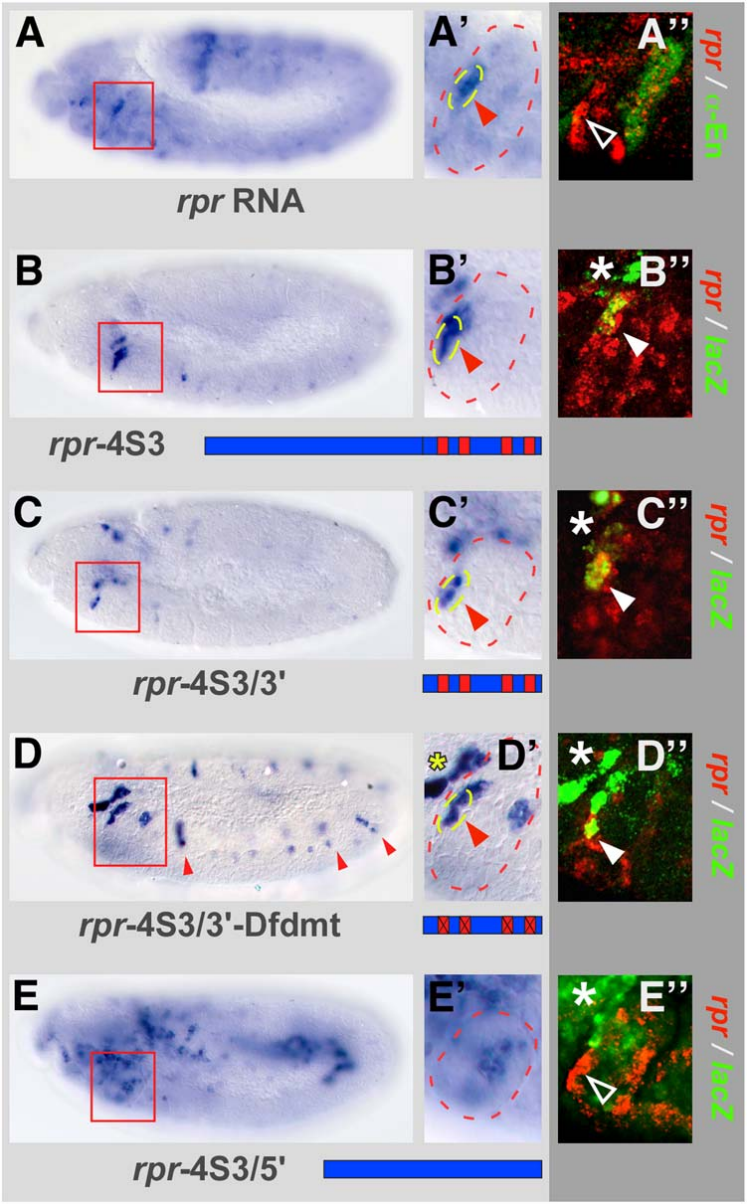
Identification of minimal Dfd response element in the *rpr* enhancer using stage 11 wild-type embryos. (A and A′) *rpr* RNA is strongly expressed in the anterior part of the maxillary segment. (A″) Double-labelling of *rpr* RNA and Engrailed (En) protein. Arrowhead marks *rpr* transcripts, mostly excluded from the posterior part of the maxillary segment (highlighted by En expression). (B and B′) *lacZ* RNA expression in the *rpr*-4S3 reporter line. (C and C′) In the *rpr*-4S3/3′ reporter line, *lacZ* expression recapitulates endogenous *rpr* transcription in the maxillary segment. (D and D′) In the *rpr*-4S3/3′-Dfdmt reporter line all four Dfd binding sites are mutated, resulting in strong *lacZ* activation in the anterior part of the maxillary segment. Small, red arrowheads in (D) indicate ectopic *lacZ* expression in trunk segments. (E and E′) In the *rpr*-4S3/5′ reporter line, *lacZ* is expressed in a broad stripe close to the posterior end. (B″ to E″) Double-labelling of *rpr* and *lacZ* RNA in the *rpr*-4S3 (B″), *rpr*-4S3/3′ (C″), *rpr*-4S3/3′-Dfdmt (D″) and *rpr*-4S3/5′ (E″) transgenic lines. The closed arrowheads in (B″ to D″) mark areas of co-localization of *rpr* and *lacZ* transcripts, the open arrowhead in (E″) marks area of *rpr* expression in the anterior part of the maxillary segment without any *lacZ* transcripts. Red boxes in (A to E) mark the maxillary segment, close-ups of which are shown in (A′ to E′). Asterisks in (B″, C″, D″ and E″) indicate area of *lacZ* expression in procephalic lobe. Blue bars in (B to E) represent different parts of *rpr* enhancer, Dfd binding sites are indicated as small red boxes.

To isolate a minimal *rpr* regulatory element able to recapitulate endogenous expression in the maxillary segment, we divided the known 674 bp long *rpr*-4S3 enhancer element [13], and analyzed *lacZ* expression driven by the resulting sub-fragments (Figure 1). We found that the 3′ part of the enhancer termed *rpr*-4S3/3′, which contained all previously defined Dfd binding sites [13], was sufficient to drive *lacZ* expression in a few cells located in the anterior part of the maxillary segment (Figure 1C′). Double-labelling experiments demonstrated co-localization of *rpr* and *lacZ* transcripts in the *rpr*-4S3/3′ line in a subset of *rpr* expressing cells (Figure 1C″).

We next tested the functional relevance of the Dfd binding sites in the *rpr*-4S3/3′ enhancer by mutational analysis. Surprisingly, we observed an increase in *lacZ* expression in the anterior part of the maxillary segment after mutating all Dfd binding sites (Figure 1D′), rather than a reduction, as observed with the same mutations in the context of the larger *rpr*-4S3 reporter [13]. Additionally, weak *lacZ* expression was observed in the anterior part of all other segments in the *rpr*-4S3/3′-Dfdmt line (Figure 1D). These findings showed that the Dfd binding sites are not exclusively used for activation, but also for repression of *rpr* transcription. Additionally, these results indicated that the overall binding site composition of the *rpr*-4S3 enhancer determines its regulatory output and that most information for repression is located in the 3′ part of the enhancer. Consistently, we found ectopic reporter gene expression in the posterior part of the maxillary segment when using the remaining 5′ part of the *rpr*-4S3 enhancer (*rpr*-4S3/5′) (Figures 1E and 1E′). While the *rpr*-4S3/3′ enhancer harbours most of the binding sites for transcriptional repression, it still has the capacity to direct region-specific activation of *rpr*, since *lacZ* expression is maintained in a few cells in the anterior part of the maxillary segment in the *rpr*-4S3/3′ line (Figure 1C′). Taken together, these results showed that the Dfd-dependent regulation of *rpr* is highly complex and that Dfd has activating and repressing activity even when acting on a small regulatory element. Thus, we decided to study the *rpr*-4S3/3′ enhancer in detail, because its reduced complexity provided a sensitive background to uncover the mechanisms of Hox target regulation *in vivo*.

### Identification of Transcription Factors Necessary for Proper *rpr* Expression

**Figure 2:**
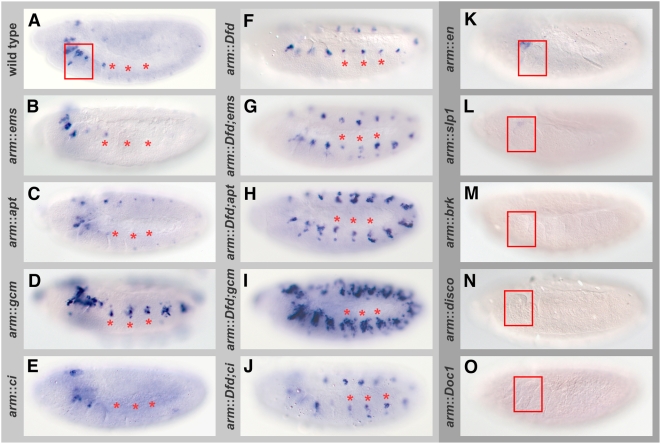
Approach to identify factors for Dfd-dependent *rpr* expression. *lacZ* RNA *in situ* hybridizations in stage 11 embryos ubiquitously mis-expressing different genes in *rpr*-4S3/3′ reporter line using the *arm*-GAL4 driver are shown: (A) *rpr*-4S3/3′ control, (B) *arm*::*ems*, (C) *arm*::*apt*, (D) *arm*::*gcm*, (E) *arm*::*ci*, (F) *arm*::*Dfd*, (G) *arm*::*Dfd;ems*, (H) *arm*::*Dfd;apt*, (I) *arm*::*Dfd;gcm*, (J) *arm*::*Dfd;ci*, (K) *arm*::*en*, (L) *arm*::*slp1*, (M) *arm*::*brk*, (N) *arm*::*disco*, (O) *arm*::*Doc1*. The screen is based on the observation that ubiquitous mis-expression of Dfd in the *rpr*-4S3/3′ line leads to ectopic *lacZ* expression in anterior part of every segment (shown in F). In (A to J) asterisks mark three spots of *lacZ* expression in trunk, box in (A, K to O) highlights the maxillary segment.

To test the effect of Dfd on the minimal *rpr*-4S3/3′ enhancer fragment, we ubiquitously expressed Dfd in the *rpr*-4S3/3′ reporter strain using the *armadillo* (*arm*)-GAL4 driver [20]. We observed specific reporter gene activation in the anterior part of every segment (Figure 2F), a result we had observed before when using the *rpr*-4S3 reporter line (data not shown). *lacZ* RNA never extended into the anterior-dorsal or anterior-ventral zone (Figures 2A and 2F). This led us to hypothesize that essential factors for the Dfd-dependent *rpr* expression are locally expressed in sub-domains of every segment, either in the anterior part if they act as activators or in the posterior, dorsal or ventral part if they act as repressors on the *rpr*-4S3/3′ enhancer.

To test this hypothesis mechanistically, we assayed 16 transcription factors, which meet the expression criteria outlined above, along with two known Dfd interactors, Apontic (Apt) and Disconnected (Disco) [21],[22] for their ability to modulate Dfd-dependent *rpr* expression (Table 1). We ubiquitously mis-expressed all factors in embryos harbouring the *rpr*-4S3/3′ reporter, and categorized them dependent on their capacity to affect reporter gene expression (Figure 2). Seven transcription factors were able to elicit the response predicted by their expression patterns (Figure S1): Apt and Glial cells missing (Gcm) activated reporter gene expression (Figures 2C and 2D), whereas Brinker (Brk), Disco, Dorsocross 1 (Doc1), En and Slp1 repressed *lacZ* expression (Figures 2K to 2O). Two of the factors identified in our screen, En and Slp1, have recently been shown to assist Hox proteins in target gene regulation [10], supporting the validity of our approach. Additionally, Disco and Apt were known to genetically and/or biochemically interact with Dfd [21],[22]. While over-expression of the activating transcription factors alone had a modest effect (Figures 2B to 2D), simultaneous over-expression with Dfd strongly enhanced reporter gene expression (Figures 2G to 2I). Similar effects were observed when we analyzed endogenous *rpr* RNA expression in these embryos (Figures S2E to S2H), suggesting that these factors are likely to function in concert with Dfd in the induction of *rpr*. Using this co-expression strategy, we identified another factor modulating *rpr* expression: Empty spiracles (Ems) enhanced the ability of Dfd to activate reporter gene expression (Figure 2G), although Ems mis-expression alone had no effect (Figure 2B). Since other candidates tested had no effect on reporter gene expression either alone or in combination with Dfd (Figures 2E and 2J), we concluded that the effects of Ems on the *rpr*-4S3/3′ enhancer are specific.

**Table 1:** Transcription factors tested for effect on *rpr*-4S3/3′ reporter gene expression.

Expression	Gene	Effect
**anterior**	*cubitus interruptus* (*ci*)	none
	*cubitus interruptus 75* (*ci75*)	none
	*glial cells missing* (*gcm*)	activating
	*empty spiracles* (*ems*)	activating
	*gooseberry* (*gsb*)	none
	*stripe* (*sr*)	none
**posterior**	*engrailed* (*en*)	repressive
	*sloppy paired 1* (*slp1*)	repressive
	*sloppy paired 2* (*slp2*)	none
**dorsal**	*Dorsocross 1* (*Doc1*)	repressive
	*Dorsocross 2* (*Doc2*)	none
	*Dorsocross 3* (*Doc3*)	none
**ventral**	*brinker* (*brk*)	repressive
	*runt* (*r*)	none
**other**	*disconnected* (*disco*)	repressive
	*apontic* (*apt*)	activating

### Role of Co-Regulatory Factors in Dfd-Dependent *rpr* Expression

**Figure 3:**
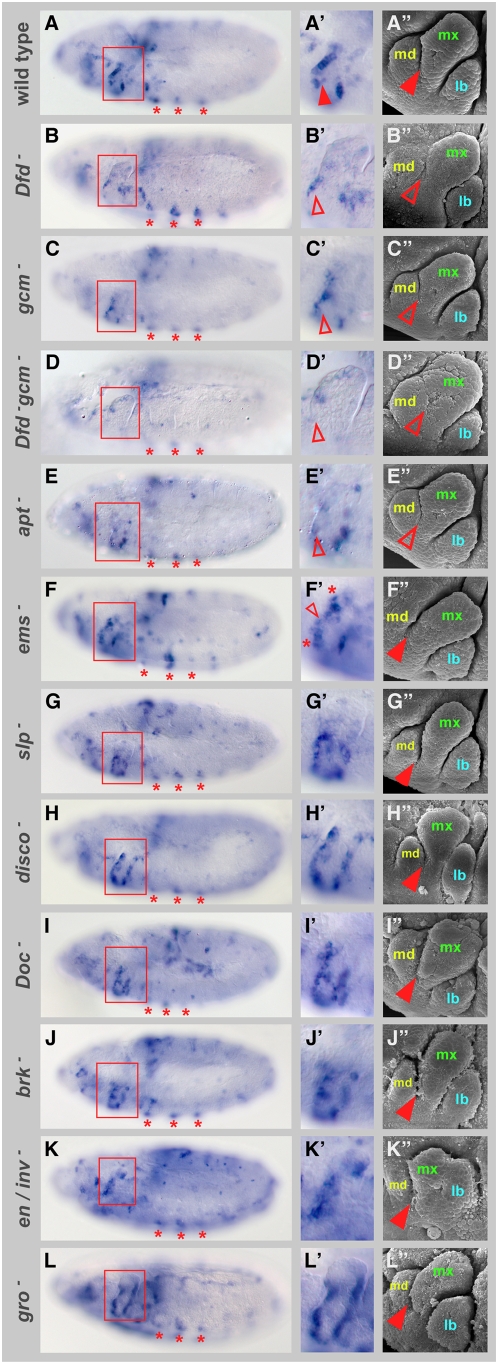
Requirement of transcription factors for *rpr* expression and development of the maxillary segment. (A–L) *rpr* RNA expression in stage 11 wild-type (A), *Dfd^w21^* (B), *gcm^N7-4^* (C), *Dfd^w21^*; *gcm^N7-4^* (D), *apt^03041^* (E), *ems^9G^/ems^7D99^* (F), *Df(2L)slp2-Δd66C* (G), *Df(1)XR14* (H), *Df(3L)DocA* (I), *brk^M68^* (J), *Df(2R)en^E^* (K) and *gro^B48^* (L) mutant embryos. To select identical stages, two criteria were used: 1) overall morphology of embryos; 2) three spots of *rpr* expression in thoracic segments characteristic for stage 11 wild-type embryos (marked by three asterisks). Red boxes in (A to L) highlight maxillary segments. (A′ to L′) Close-up of maxillary segments in respective mutants. In *gcm^N7-4^* and *apt^03041^* mutants, *rpr* expression is reduced (C′ and E′), in *Dfd^w21^*; *gcm^N7-4^* double mutants expression is lost (D′) (open arrowhead). In *ems^9G^/ems^7D99^* mutants, levels of *rpr* transcripts are reduced in middle part of anterior *rpr* expression area (small open arrowhead), in ventral-anterior and dorsal-anterior part *rpr* transcript levels are increased (highlighted by asterisks). In embryos mutant for repressing transcription factor genes, cells ectopically expressing *rpr* are observed in various parts of the maxillary segment (G′ to K′). In *gro^B48^* mutants, *rpr* expression in anterior and posterior parts is increased (L and L′). (A″ to L″) Scanning electron micrographs of gnathal segments of late stage 12 embryos of respective mutants. Mandibular (md), maxillary (mx) and labial (lb) segments are indicated in this panel. In mutants for the activating transcription factor genes, the boundary between the maxillary and mandibular segments is reduced or abolished (C″ to E″) (open arrowhead), reminiscent to the effects seen in *Dfd* mutants (B″), in mutants for the repressing transcription factor genes this boundary is unaffected (G″ to K″) (closed arrowhead).

To test whether the factors identified are necessary for proper *rpr* transcription in the maxillary segment, we analyzed *rpr* transcripts in embryos mutant for the individual transcription factor genes (Figure 3). Additionally, we studied the morphology of the gnathal lobes, in particular the boundary between the maxillary and mandibular segments, since it is known that maintenance of this boundary depends on proper *rpr* activity [13]. In mutant embryos of two activators, *gcm* and *apt*, we observed a reduction of *rpr* expression in the anterior part of the maxillary segment (Figures 3C′ and 3E′) and a slight fusion of the maxillary and mandibular segments (Figures 3C″ and 3E″). The latter phenotype was not as pronounced as in *Dfd* mutants (Figure 3B″), which exhibit a strongly reduced *rpr* expression in the anterior part of the maxillary segment (Figure 3B′; [13]). In *ems* mutants, the maxillary-mandibular boundary developed normally (Figure 3F″). Here, *rpr* transcript levels were only reduced in the middle part of the anterior *rpr* expression domain, whereas dorsally and ventrally to this area *rpr* transcript levels were elevated (Figure 3F′). As reported previously [23], a loss of the mandibular segment was observed in *ems* mutant embryos (Figure 3F″). To test the interactions between Dfd and the activating transcription factors genetically, we extended our studies to *Dfd gcm* double mutants. In these embryos, *rpr* expression and the formation of the maxillary-mandibular boundary were completely lost (Figures 3D′ and 3D″). This result not only confirmed an important role of the activating factors for *rpr* expression and the maintenance of the segment boundary, but also suggested that Dfd and Gcm act independently.

In embryos mutant for the repressing transcription factor genes, we observed ectopic *rpr* expression in the maxillary segment, primarily in the central or posterior part (Figures 3G′ to 3K′), showing that the factors are involved in repressing *rpr* transcription. The de-repression of *rpr* transcription in only a few cells suggested that repression of *rpr* transcription is redundant. Thus, we aimed to analyze *rpr* expression in embryos mutant for multiple transcription factor genes with repressive function. However, due to lethality, we were not able to generate any double mutant combination. To circumvent this problem, we made use of the fact that the activity of Slp1, Brk and En is dependent on the transcriptional co-repressor Groucho (Gro) [24],[25],[26],[27]. We hypothesized that *gro* mutant embryos should behave similarly to a triple knock-out of the repressor genes with regards to *rpr* regulation. Consistently, the number of cells ectopically expressing *rpr* was further increased in *gro* mutants when compared to the single mutants. The effect was most pronounced in the posterior part of the maxillary segment (Figures 3L and 3L′). Phenotypic analysis of the repressor mutants revealed that the maxillary-mandibular boundary was not affected (Figures 3F″ to 3K″). This was consistent with largely unchanged *rpr* transcription in the anterior part of the maxillary segment in all mutants for repressive transcription factors (Figures 3F′ to 3K′). Nevertheless, the overall morphology of the gnathal lobes in these mutants was abnormal (Figure 3).

**Figure 4:**
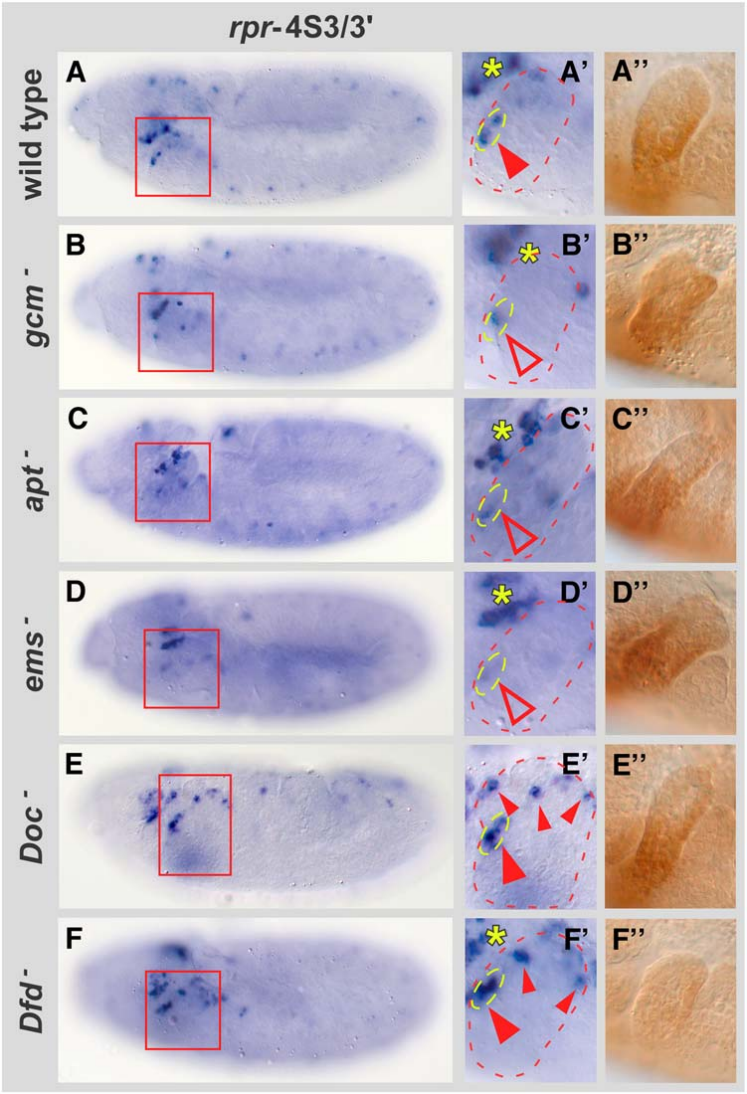
Co-regulatory transcription factors are required for proper *lacZ* expression in stage 11 *rpr*-4S3/3′ reporter line. (A to F) In *rpr*-4S3/3′ reporter line, β-galactosidase expression in the following genetic backgrounds is shown: (A) *rpr*-4S3/3′ control, (B) *gcm^N7-4^*, (C) *apt^03041^*, (D) *ems^9G^/ems^7D99^*, (E) *Df(3L)DocA*, (F) *Dfd^w21^* mutant embryos. Red boxes in (A to F) highlight maxillary segments. (A′ to F′) Close-up of maxillary segments in respective mutants. The yellow asterisks in (A′, B′, C′, D′ and F′) mark expression of *lacZ* in procephalic lobes. (A″ to F″) Dfd protein expression in the respective genotypes. Note that although the morphology of the maxillary segment is changed, the expression domain and intensity of Dfd protein in the respective mutants (B″ to E″) is very similar to wild-type Dfd protein expression (A″).

To exclude the possibility that changes in Dfd expression cause the modifications in *rpr* activity and boundary formation observed in the transcription factor mutants, we analyzed Dfd protein localization in these embryos. Since Dfd expression was always unaffected (Figures 4B″ to 4E″), we concluded that the factors identified do not act upstream, but in parallel to Dfd in the regulation of *rpr*. In addition, we could rule out cross-regulatory effects between *gcm* and Slp [28] in the maxillary segment (data not shown).

After having shown a functional relevance for the identified transcription factors, we studied their contribution to *rpr* expression in the context of the *rpr*-4S3/3′ enhancer element, since gene expression is often resistant to the modulation of individual trans-acting factors acting on large and redundant enhancers. We observed a strong reduction of *lacZ* RNA in embryos mutant for the activating transcription factors (Figures 4B′ to 4D′). This result suggests that all three factors play important roles in *rpr* activation and that they act on regulatory elements contained within the *rpr*-4S3/3′ enhancer. In embryos mutant for repressing transcription factors, ectopic *lacZ* activation was observed only in some maxillary cells, as shown for the *Doc* mutant (Figure 4E′), suggesting that transcriptional repression is redundant even at the level of the *rpr*-4S3/3′ enhancer. Consistent with the binding site mutations (Figure 1D′), *lacZ* was strongly activated in the anterior part (but also in other parts) of the maxillary segment in *Dfd* mutants (Figures 4F and 4F′). These results confirmed that in the *rpr*-4S3/3′ context Dfd acts primarily as a repressor, and suggested that full repression is achieved by the combined action of Dfd and additional transcription factors.

### Direct Interaction of Co-Regulatory Factors with Minimal *rpr* Enhancer

**Figure 5:**
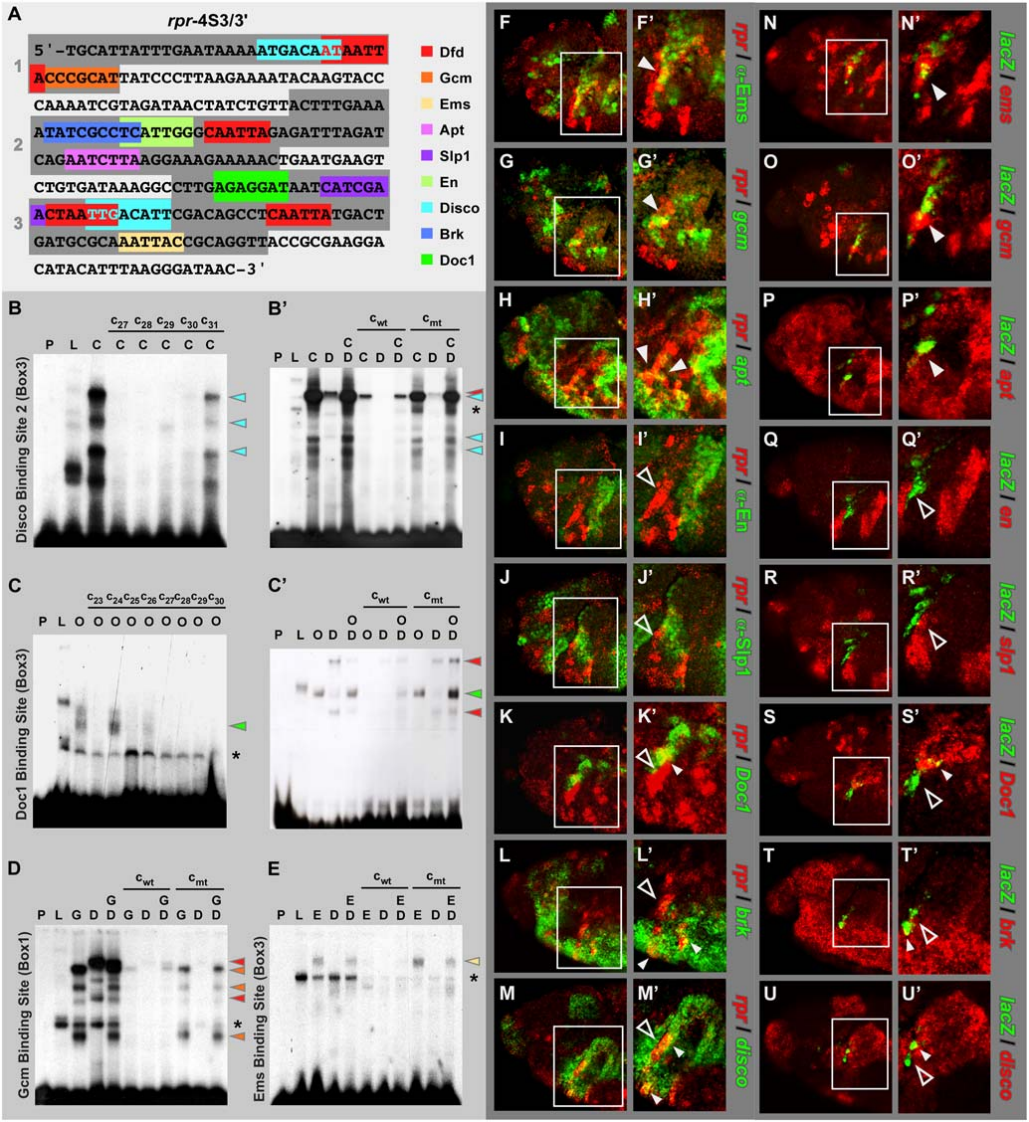
Co-regulatory transcription factors directly interact with *rpr*-4S3/3′ enhancer. (A) Sequence of *rpr*-4S3/3′ enhancer fragment with binding sites for Dfd (shown in red) and all identified co-regulatory transcription factors (highlighted in different colours) is shown. Conserved regions 1 to 3 within the *rpr*-4S3/3′ enhancer are highlighted as dark grey boxes. (B and C) EMSAs for mapping of Disco binding site 2 (B) and Doc1 binding site (C) using box 3 as shift probe. EMSA was performed using no protein (P), translation lysate only (L), lysate with Dfd protein (D), lysate with Disco protein (C) and lysate with Doc1 protein (O). c_27_ to c_31_ in (B) and c_23_ to c_30_ in (C) represent consecutive competitor oligonucleotides with their middle base-pairs mutated. Competition experiments revealed that sequences mutated in the oligonucleotide c_31_ include binding site for the Disco protein (B), whereas oligonucleotides c_24_ include binding site for the Doc1 protein (C). The turquoise and green arrowheads indicate specific DNA-protein complexes containing either Disco or Doc1 protein, respectively. (B′, C′, D, E) EMSAs using no protein (P), translation lysate (L), lysate with Dfd protein (D), Doc1 protein (O), Gcm protein (G), Ems protein (E) and lysate with Dfd protein (D). To test specificity of binding of the proteins to the DNA fragments, competitor oligonucleotides for the mapped binding sites were used either in their wild-type (c_wt_) or mutant (c_mt_) sequence versions. Red arrowheads indicate specific DNA-protein complexes containing Dfd protein, turquoise, green, orange or light-yellow arrowheads indicate specific DNA-protein complexes containing Disco, Doc1, Gcm or Ems proteins, respectively. Note that in all competitor oligonucleotides only binding site sequences for co-regulatory transcription factors are mutated, but not for Dfd binding sites. (F to U′) Protein or RNA co-localization of co-regulatory transcription factors and *rpr* (F to M′) or *lacZ* RNA (N to U′) in head of stage 11 wild-type (F to M′) or *rpr*-4S3 reporter line (N to U′) embryos. Boxes mark maxillary segment with *rpr* or *lacZ* RNAs present in anterior part. In (F′ to M′ and N′ to U′) close-ups of maxillary segments are shown. Co-localization of *rpr* or *lacZ* RNAs and co-regulator RNA is observed in individual cells for Doc1, Brk, Disco (K′ to M′ and S′ to U′; small, closed arrowheads). Closed arrowheads mark cells co-expressing *rpr* and *lacZ* RNAs and RNA or protein of activating co-regulators, open arrowheads highlight areas of *rpr* or *lacZ* transcription and missing expression of repressing co-regulators in anterior part of maxillary segments. Asterisks in (B′, C, D and E) indicate complexes with lysate protein seen also in the controls.

We next addressed whether the identified factors act directly in the regulation of *rpr* in the maxillary segment. Thus, we studied the expression of *rpr* and all factors with cellular resolution using double-labelling experiments. *rpr* transcripts always co-localized with the activating transcription factors, whereas they were mostly excluded from cells positive for the repressing transcription factors (Figures 5F′ to 5M′). We obtained the same result when *lacZ* transcript distribution and expression of the co-regulatory factors in the *rpr*-4S3 reporter line were analyzed (Figures 5N to 5U). *rpr* transcripts and the activators co-localized in distinct sub-domains of the *rpr* expression zone: Ems in the dorsal most, Gcm in the middle and Apt in the ventral most part (Figures 5F′ to 5H′). This suggests that individual activating factors are responsible for *rpr* transcription in distinct cells in the anterior part of the maxillary segment and that their combined activity is required for the expression of *rpr* in its complete domain.

To further test whether the identified transcription factors are directly involved in the expression of *rpr* on the mechanistic level, we mapped transcription factor binding sites in the *rpr*-4S3/3′ enhancer using phylogenetic footprinting [29],[30],[31]. Using species-specific *rpr* RNA probes, we could show that *rpr* was expressed specifically in the anterior part of the maxillary segment in all seven *Drosophila* species chosen (Figure S3). We then isolated the *rpr*-4S3/3′ enhancer fragment from all species and after aligning the sequences using the TCoffee algorithm [32],[33], we identified three highly conserved boxes, which contained all four Dfd binding sites previously characterized (Figure S3). Furthermore, we found known consensus binding motifs for three of the eight factors within the conserved regions (Figure 5A, Figure S3) [10],[34],[35],[36].

To molecularly test direct binding of all factors identified in the *rpr* enhancer, we performed electrophoretic mobility shift assays (EMSA). We found that all eight transcription factors interact with conserved regions in the *rpr*-4S3/3′ enhancer *in vitro* (Figure 5, Figure S4). To define the DNA sequences necessary for this interaction, systematic competition experiments using overlapping and mutated oligonucleotides for each conserved box were performed. This analysis allowed us to confirm the published Gcm consensus sequence -ACCCGCAT- [37] (Figure 5D), which is located directly adjacent to Dfd binding site 1 in the *rpr*-4S3/3′ fragment (Figure 5A, Table 2). Similarly, En and Slp1 binding sites are found in close proximity to Dfd binding sites 2 and 3 (Figure 5A). Our EMSA analysis uncovered that the assisting factors Apt, En, Slp1 and Brk interact with binding sites slightly divergent from published consensus sequences (Figure 5, Figure S4, Table 2) [10],[38],[39],[40]. Finally, we identified unknown DNA binding sequences for the assisting factors Doc1 and Disco (Figure 5B, C, Figure S4, Table 2). Our competition experiments revealed that Disco protein interacts with two binding sites in the *rpr*-4S3/3′ enhancer (Figure 5B, Figure S4), which share an invariant five nucleotide core motif, -TGACA- (Figure 5A, Table 2).

**Table 2:** Published and mapped binding sites for all transcription factors within the *rpr*-4S3/3′ Hox response element.

Transcription factor	Mapped DNA binding sites	Published DNA binding sites	Reference
**Gcm**	**ACCCGCAT**	( **A**/G)**CCCGCAT**	Akiyama et al., 1996
**Apt**	AA**TCTTA**	(A/G)T**TC**(C/**T**)(A/**T**)**A**T(T/A)(G/A)GA(A/T)(T/C)	Liu et al., 2003
**Ems**	A**ATTA**C	AAXTX**TAAT**GACA	Taylor, 1998
**En**	**TCATT**GG	**TCATT**C	Gebelein et al., 2004
**Slp1**	**CATCGAA**	GGTGTGTTGA**CATCGAA**GA	Yu et al., 1999
**Brk**	TAT**CGCC**T**C**	( **C**/T)**GCC**A(G/**C** )	Sivasankaran et al., 2000
**Doc1**	AGAGGAT	-	-
**Disco**	A**TGACA**AT	-	-
	T**TGACA**TT		

Bold letters highlight identical nucleotides within the *rpr*-4S3/3′ fragment and published consensus sequences.

**Figure 6:**
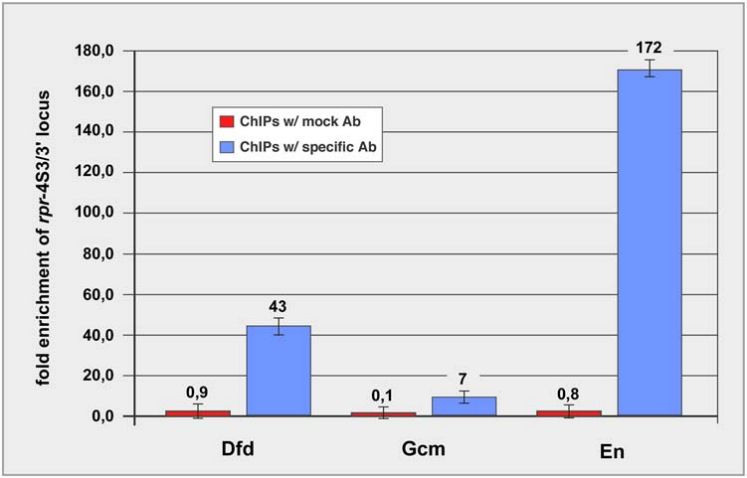
Chromatin immuno-precipitation (ChIP) for Dfd, En and Gcm confirms interaction with *rpr*-4S3/3′ enhancer *in vivo*. Specific enrichment of binding sites within the *rpr*-4S3/3′ enhancer was assayed by quantitative real-time PCR and compared to negative control locus. All ChIPs performed with specific antibodies (blue) yield at least 7-fold enrichment over the negative control, precipitations with mock antibodies (red) yield no enrichment (ratios below 1). Fold enrichment were normalized against input chromatin sample and to negative control region for primer normalization (for details: see Materials and Methods).

To test if the identified target sequences are directly bound by the factors *in vivo*, we analyzed the ability of the transcription factors to bind to the *rpr*-4S3/3′ enhancer in the context of chromatin. To this end, we performed chromatin immuno-precipitation assays (ChIPs) for all factors for which functional antibodies were available. A significant enrichment of the *rpr*-4S3/3′ locus was observed using Dfd, Gcm and En antibodies (Figure 6). These results demonstrated that Dfd, Gcm and En directly interact with specific target sequences in the *rpr*-4S3/3′ enhancer *in vivo*.

Our EMSA experiments also revealed that all factors are able to bind independently of Dfd to the *rpr*-4S3/3′ enhancer, since formation of protein complexes between Dfd and the co-regulatory transcription factors was not observed (Figures 5B to 5E, Figure S4). Consistently, GST pulldown and yeast-two hybrid assays did not provide any evidence for direct interactions of the identified transcription factors with Dfd (data not shown). To exclude the possibility that more than two factors are required for complex formation on DNA, which has been shown before for other HREs [41], we performed EMSA experiments using a mixture of three transcription factors and conserved box 1 as probe. In this region the binding sites for Disco, Dfd and Gcm lie in close proximity, which is considered a requirement for cooperative binding [41]. However, we did not observe a higher-order complex when incubating conserved box 1 with extracts containing all three transcription factor proteins (data not show). Thus, we conclude that these regulators do not bind the *rpr*-4S3/3′ enhancer in a cooperative manner.

### Contribution of Co-Regulatory Factors to Dfd-Dependent *rpr* Expression

**Figure 7:**
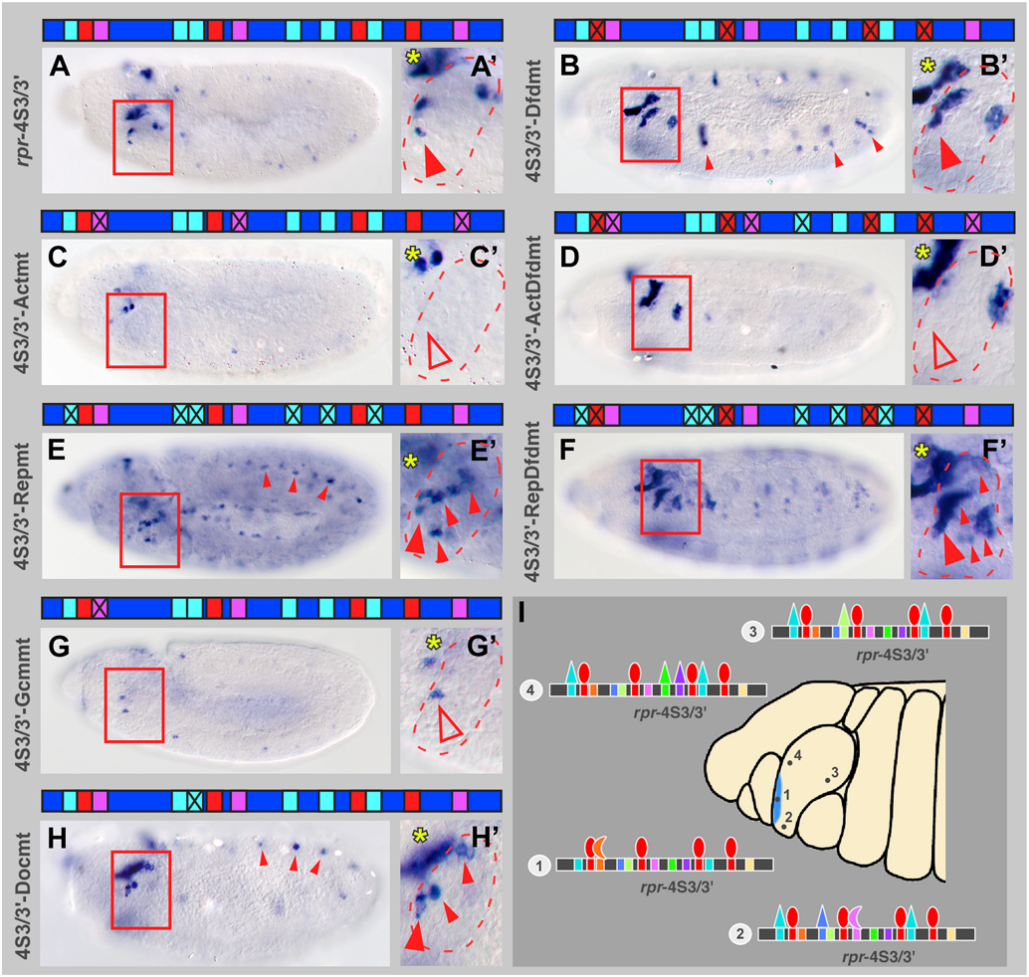
Binding sites for co-regulatory transcription factors are required for *rpr* enhancer activity in stage 11 embryos. (A and A′) β-galactosidase is expressed in anterior part of maxillary segment in *rpr*-4S3/3′ reporter line. Closed, red arrowhead marks anterior part of maxillary segment. (B and B′) In the *rpr*-4S3/3′-Dfdmt reporter, with all Dfd binding sites mutated, *lacZ* expression is increased in anterior part of maxillary segment (closed, red arrowhead). *lacZ* expression is ectopically induced in anterior part of every segment (small, closed arrowheads). (C and C′) In the *rpr*-4S3/3′-Actmt line, with all sites for activating co-regulators mutated, reporter gene expression in maxillary segment is lost (open arrowhead). (D and D′) In the *rpr*-4S3/3′-ActDfdmt reporter, with all Dfd binding sites and sites for activating co-regulators mutated, *lacZ* expression in the anterior part is lost (open arrowhead). (E and E′) In the *rpr*-4S3/3′-Repmt line, with all sites for repressing co-regulators mutated, additional cells in maxillary segment express reporter gene. In rest of embryo, *lacZ* expression is ectopically induced (small, closed arrowheads). (F and F′) In the *rpr*-4S3/3′-RepDfdmt reporter, with all Dfd binding sites and sites for repressing co-regulators mutated, *lacZ* expression in anterior and posterior parts is strongly induced (closed arrowheads). (G and G′) In stage 11 embryos of *rpr*-4S3/3′-Gcmmt line, with the Gcm binding site mutated, reporter gene expression in anterior part of maxillary segment is reduced (open arrowhead). (H and H′) In the *rpr*-4S3/3′-Docmt line, with the Doc1 binding site mutated, reporter gene expression is observed in additional cells in maxillary segment. In the rest of the embryo, *lacZ* expression is ectopically induced (small, closed arrowheads). (I) Model of *rpr* regulation through the *rpr*-4S3/3′ enhancer. Expression of *rpr* in the anterior part of the maxillary segment (highlighted in blue) is achieved through a combinatorial interaction of the Hox protein Dfd and co-regulatory transcription factors (represented as different-coloured triangles) to specific binding sites in the *rpr*-4S3/3′ enhancer. Each cell of the maxillary segment expresses different combinations of Dfd and the co-regulatory transcription factors, which is reflected in a cell type-specific occupancy of the *rpr*-4S3/3′ enhancer, as shown exemplarily for four different cells (marked 1 to 4). According to the model, the decision whether *rpr* transcription is activated or repressed in individual maxillary cells depends on the nature and combination of regulatory factors interacting with the *rpr*-4S3/3′ enhancer. Boxes in (A to F) highlight maxillary segments, yellow asterisks in (A′ to H′) mark *lacZ* expressing cells in procephalic lobes. *rpr*-4S3/3′ enhancer in (A to H) is represented as blue bar, Dfd binding sites as red, sites for activating co-regulators as pink and sites for repressing co-regulators as turquoise boxes.

We next tested the importance of the identified DNA binding sites for Dfd-dependent *rpr* expression in the embryo. To this end, we mutated all sites for the activating or repressing transcription factors in the *rpr*-4S3/3′ element and analyzed reporter gene expression. We found that *lacZ* expression was abolished, when binding sites for all three activating factors, Gcm, Apt and Ems, either alone or in combination with the Dfd binding sites, were mutated (Figures 7C and 7D). These results show that these factors, independently of Dfd, are responsible for activation of the *rpr*-4S3/3′ enhancer element in the anterior part of the maxillary segment. A reduction of *lacZ* expression was even observed when a single binding site for an activating factor was mutated (Figures 7G and 7G′), further suggesting that the combined action of all three factors is required for activation of the *rpr*-4S3/3′ element. In embryos carrying the *rpr*-4S3/3′ enhancer fragment with all sites for the repressing transcription factors mutated, we observed ectopic reporter gene expression in some, but not all maxillary cells (Figure 7E′). Additionally, *lacZ* was expressed throughout the embryo (Figure 7E). Finally, when all Dfd and repressor sites were mutated, *lacZ* transcription was activated in even more cells of the maxillary segment (Figure 7F and 7F′), confirming that Dfd acts in parallel to the repressing factors. Here, reporter gene expression was strongly increased in the anterior and posterior part of the maxillary segment (Figure 7F′). However, we never observed *lacZ* expression throughout the whole maxillary segment, suggesting that there are additional, unidentified repressors of *rpr* expression. When single binding sites for repressing transcription factors were mutated, as shown for the Doc1 binding site (Figure 7H′), ectopic reporter gene expression in the maxillary segment was observed, confirming the importance for the direct interaction of repressors with *rpr*-4S3/3′ element.

## Discussion

We have shown that eight transcriptional regulators, Apt, Gcm, Ems, En, Slp1, Brk, Doc1 and Disco, are required in addition to the Hox protein Dfd to properly regulate the expression of the apoptosis gene *reaper* in a specific subset of cells of the maxillary segment. The finding that such a large number of structurally unrelated transcription factors with important and diverse functions during differentiation and cell-type specification processes assist Dfd in the regulation of the *rpr*4S3/3′ enhancer element was surprising. For example, Gcm is one of the major regulators of glial cell differentiation, consistent with its expression in glial precursor cells during embryogenesis [42],[43]. Two other factors, Brk and Doc1, are both known to play important roles in the Dpp/TGF-β signalling pathway: Brk, a genuine transcriptional repressor [24], acts as a negative regulator of Dpp-dependent genes [44],[45],[46], whereas Doc1, one of the three genetically redundant Dorsocross transcription factors required for amnioserosa development in *Drosophila* [47], is a direct target of the Dpp pathway [48]. Furthermore, Apt and Disco have previously been found in genetic screens designed to identify modifiers/interactors of Dfd [21],[22]. Mutations in these genes, which are expressed in the gnathal segments [21],[22], result in severe malformations of structures derived from these segments, similarly to the defects observed in *Dfd* mutants. And finally, we have identified three factors known to be critically involved in patterning the A/P axis as important regulators of *rpr* expression: the gap-like segmentation gene product Ems, which is important for patterning embryonic head structures [49],[50], and two segment polarity factors, En and Slp1. Taken together, our findings suggest that proper spatio-temporal Hox target gene regulation is achieved by the combined action of multiple transcriptional regulators: the Hox proteins themselves and a large number of structurally diverse transcription factors. Although the interaction with additional transcription factors has been reported before [11],[14],[51], the finding that a multitude of diverse factors is required to regulate the activity of a small HRE adds a new layer of complexity to the mechanisms of Hox target gene regulation.

Our results not only demonstrate that the newly identified factors are functionally involved in *rpr* regulation, but also show mechanistically that they contribute to localized *rpr* activation by direct interactions with specific DNA sequences located in the *rpr*4S3/3′ enhancer element both *in vitro* and *in vivo*. Additionally, all factors very likely bind to their target sequences independently of Dfd. Thus, our findings support and significantly extend recent observations: the repression of the Hox target *sal* in the haltere requires the direct interaction of the Hox protein Ubx and two Dpp downstream effectors, Mad and Med, with adjacent binding sites in the *sal*1.1 CRE [18]. As in our case, no evidence for a direct cooperative interaction of the assisting transcription factors with the Hox protein was detected. Thus, we postulate that the Hox-dependent regulation of *rpr* expression is, as in the case of *sal* repression by Ubx, achieved through combinatorial regulation, in which two or more regulatory proteins bind to nearby sites, but not necessarily to each other [18].

While combinatorial regulation of gene expression has been extensively studied for diverse transcription factors [52],[53], our results shed new light on the mechanisms of Hox target gene regulation. Previously, much attention focused on the Hox cofactors Extradenticle (Exd) and Homothorax (Hth), which allow Hox proteins to differentially recognize and select some of their target genes through cooperative complex formation [54],[55],[56],[57]. Although Hox cofactors like Exd can explain why different Hox proteins have different DNA binding specificities, the interaction with these factors was not able to explain how broadly expressed Hox proteins are able to affect target gene expression in only a subset of cells. One of the major reasons for that is that Exd and Hth, the only well-known Hox cofactors, are expressed throughout the embryo and interact promiscuously with most Hox proteins. In addition, studies on Exd and Hth revealed that Hox target gene regulation more or less inevitably includes complex formation between Hox proteins and assisting co-regulatory factors. We and others have now shown that the ability of a broadly expressed Hox protein to regulate a target gene in a proper spatial and temporal context is achieved by the Hox-independent recruitment of context-specific transcription factors to cis-regulatory sequences present in compact HREs [18]. Based on our findings, we now suggest that a large number of transcription factors could dictate the transcriptional output in combination with the respective Hox protein by binding selectively and independently to cis-regulatory sequences within HREs of target genes (Figure 7I). Since every cell has a unique combination of transcription factors, the combinatorial interactions for the broadly expressed Hox proteins are almost limitless in such a scenario, accounting for the precise modulation and fine-tuning of Hox target gene regulation, even on the level of the individual cell. Additionally, this model can explain how Hox proteins can act as repressors in one context and as activators in another, because the combined transcriptional output is dependent on the regulatory activity of all transcription factors assembled on a HRE (Figure 7I). There are several lines of evidence that support this model: first, the invariable ectopic activation of Hox downstream genes in spatially restricted domains of every segment when upstream Hox proteins are ubiquitously mis-expressed [8], and second, the accumulation of binding sites for additional transcription factors in enhancers predicted to be controlled by Hox proteins [8]. Alternatively, it seems also possible that Dfd regulates the expression of its target gene *rpr* only in some maxillary cells, while the novel co-regulatory factors mediate regulation in other maxillary cells. However, since Dfd protein is present in all cells of the segment, the first model seems more likely. Irrespective of the mechanism used by Dfd, it will be essential to study the architecture of HREs, with a special focus on the binding site composition of these enhancers and the diverse factors binding to them to further advance our understanding of Hox target gene regulation *in vivo*.

It has been argued before that context-specific transcription factors assisting Hox proteins in target gene regulation are not likely to act as transcriptional repressors or activators themselves, but rather recruit co-repressors and/or co-activators, and thereby dictate the transcriptional output imprinted in HREs [18],[58]. Our finding of the co-repressor Groucho playing a role in the Dfd-dependent repression of *rpr* transcription now substantiates this hypothesis, since three of the factors identified in our work, En, Slp1 and Brk, are known to require interactions with the Groucho co-repressor for the transcriptional repression of some of their downstream genes [24],[25],[59]. Interestingly, at least two other co-regulatory transcription factors identified in this work are also known to interact with co-activators/co-repressors: Apt is able to recruit the transcriptional co-activator Multiprotein bridging factor 1 (MBF1), thereby mediating Apt-dependent transcriptional activation [35]. Disco has been found in a yeast-two-hybrid screen to interact with the well-known co-repressor C-terminal Binding Protein (CtBP) [60], which is also recruited by Brk to repress some Dpp-responsive genes [24]. Since there is accumulating evidence that co-repressors, like CtBP, execute their function on transcriptional regulation through chromatin modification [61],[62], it is tempting to speculate that Hox proteins regulate their target genes also by epigenetic control mechanisms. In summary, the multitude of potential regulatory mechanisms used by Hox proteins might be the reason why it has been impossible to fully elucidate how Hox proteins mediate their function with high specificity and precision *in vivo*. In the light of recent advances, it now seems likely that the mechanisms leading to functional specificity of Hox proteins are dependent on the cellular context, the composition of the target enhancer element and the identity of the individual Hox protein. Thus, one could argue that during evolution Hox proteins have undergone individualization in trans, as well as sequence diversification in cis.

## Materials and Methods

### *Drosophila* Genetics

*D. simulans*, *D. yakuba*, *D. erecta* and *D. mojavensis* were obtained from the Tucson *Drosophila* Stock Center. The *D. melanogaster* strain used was Oregon-R. *apt^03041^*, *ems^7D99^*, *gcm^N7^* ^-4^, *Df(2R)en^E^*, UAS-*disco*, UAS-*gcm* strains were obtained from the Bloomington Stock Center; *gro^B48^* line, P. Heitzler [63]; *ems^9G^* flies, W. McGinnis [38]; *brk^M68^*, UAS-*brk* lines, S. Roth [45]; *Df(1)XR14* flies, H. Saumweber [64]; *Df(3L)DocA*, UAS-*Doc1* strains, M. Frasch [48]; *Df(2L)slp2-Δd66C* strain, W. Gehring [65]; UAS-*en* flies, I. Guerrero [66]; UAS-*ems* line, H. Jäckle [67]; UAS-*slp1* flies, M. Leptin [68]; UAS-*apt* strain, R. Schuh [69]; UAS-*ci* flies, T. Kornberg [70]. The following lines are described in Hueber et al. (2007): *Dfd^w21^*, *Dfd^r11^*, *arm*-GAL4, *prd*-GAL4, UAS-*Dfd*, UAS-*lacZ*. The following green balancer lines were used: *Dr^Mio^*/TM3*Sb*[*twi*::2xEGFP], *In(2LR)Gla wg*-Gla/Cyo[*twi*::2xEGFP] , *N*/FM7c[*twi*::2xEGFP].

### Plasmids

cDNAs were obtained from: *disco*, *Drosophila* Genomics Resource Center (GH27656), *gcm* cDNA, G. Technau [71], *brk* cDNA, C. Rushlow [45],[72], *Doc1* cDNA, M. Frasch [48], *apt* cDNA, R. Schuh [69]. Mutations in the *rpr*-4S3/3′ fragment were created by site-directed mutagenesis via two-step PCR or the QuickChange Multi Site-directed Mutagenesis Kit (Stratagene). Primer sequences are available upon request. All products were cloned, sequenced, and shuttled into pH-Pelican plasmid [73]. All transgenic fly lines were generated by the BestGene *Drosophila* Embryo Injection Service. At least three independent lines were analyzed for expression levels. *rpr* coding regions from different *Drosophila* species were PCR amplified with specific primers, cloned and sequenced.

### Histology and Scanning Electron Microscopy

*In situ* hybridization and immunochemistry were performed as described [74],[75]. Fluorescent RNA / protein double labelling and fluorescent duplex *in situ* hybridizations were done as described previously [13],[76]. Probe detection was done using the TMR and Fluorescein TSA Amplification kits from PerkinElmer (Waltham, MA). Antibodies were: rat anti-Ems (1∶200), U. Walldorf; mouse anti-En (mAb4D9) (1∶200), Developmental Studies Hybridoma Bank (Iowa, University); guinea pig anti-Slp (1∶200), J. Jäckle; anti-DIG POD, Roche (Penzberg, Germany); anti-mouse AlexaFluor 488, anti-guinea pig AlexaFluor 488 and anti-rat AlexaFluor 488, Molecular Probes. All fluorescent images were taken at Zeiss LSM510 META confocal microscope. SEM analysis was performed as described in Lohmann et al. (2002).

### Electrophoretic Mobility Shift Assays

EMSA was performed as described previously (Lohmann et al., 2002). For the zinc finger transcription factor Disco, 1 mM ZnSO_4_ was included in the binding reaction. For the mapping of binding sites, the ability of all eight transcription factors to interact with conserved boxes 1 to 3 was tested, to define the interaction domains on the *rpr*-4S3/3′ fragment. Subsequently, if known binding sites were present within the conserved boxes, competition experiments were performed to test if these sites are necessary for binding. For all factors with unknown binding sites, systematic competition experiments using overlapping and mutated oligonucleotides covering the binding region were performed. All oligonucleotide sequences used for these experiments can be obtained upon request.

### Chromatin Immuno-Precipitation (ChIP)

ChIP experiments were performed as described previously at www.flychip.org. Four independently staged wild-type embryo populations were collected, chromatin samples were prepared from 5 to 9.5 hr embryo collections. Antibodies used were the following: guinea pig anti-Dfd, guinea pig anti-IgGs (gift from H. Schwarz, MPI Tuebingen), mouse anti-En, mouse anti-LacZ (Invitrogen), rat anti-Gcm (gift from M. Wegner, University Erlangen) and rat anti-GFP (Invitrogen). A dilution of 1∶500 was used for the anti-Dfd, anti-En and anti-Gcm antibodies, the mock antibodies were used at equivalent protein concentrations. Amplification of the *rpr*-4S3/3′ and an unrelated, non-coding control locus were analyzed by quantitative real-time PCR in technical triplicates using at least two biological replicates. Precipitates were normalized to input DNA (i.e., sonicated, pre-ChIP DNA) and compared to the non-coding negative control region. A PCR efficiency of 1.8-fold amplifications per cycle was assumed. PCR primer sequences can be obtained upon request.

## Supporting Information

Figure S1 Expression patterns of the identified transcription factors in stage 11 wild-type embryos. For the following genes, antibody stainings are shown: *ems* (B), *en* (C) and *slp1* (E). Due to the unavailability or inactivity of antibodies, *in situ* hybridizations for the following genes are shown: *gcm* (A), *apt* (D), *Doc1* (F), *brk* (G) and *disco* (H). Boxes in (A to H) highlight the maxillary segment.
(2.53 MB TIF)

Figure S2 Identified transcription factors modulate *rpr* expression when mis-expressed. *rpr* RNA in situ hybridizations in stage 11 embryos with the following genotypes are shown: (A) wild type, (B) *prd*::*Dfd*, (C) *prd*::*ems*, (D) *prd*::*Dfd;ems*, (E) *prd*::*apt*, (F) *prd*::*Dfd;apt*, (G) *prd*::*gcm*, (H), *prd*::*Dfd;gcm*. Co-expression of Ems, Apt and Gcm with Dfd enhances Dfd-dependent ectopic *rpr* induction (D, F and H), whereas Gcm is able to ectopically induce *rpr* expression alone. To select identical stages, three characteristic spots of *rpr* expression in the thoracic segments normally seen in stage 11 wild-type embryos (marked by three asterisks) were used. In (B to H) one spot of ectopic *rpr* expression at the very posterior end in the *prd*-GAL4 over-expression embryos is marked by a red arrow, the blue box outlines an additional stripe of *rpr* RNA expression in the T3 primordium.
(6.46 MB TIF)

Figure S3 Identification of conserved regulatory elements in the *rpr*-4S3/3′ enhancer by phylogenetic footprint analysis. Upper: *rpr* RNA expression in stage 11 embryos of different *Drosophila* species used for the phylogenetic footprint analysis. *In situ* hybridization experiments with species-specific probes show that *rpr* is expressed in the anterior part of all five *Drosophila* species (*D. melanogaster*, *D. simulans*, *D. yakuba*, *D. erecta*, *D. mojavensis*). The red boxes highlight the maxillary segment. Bottom: Alignment of the *rpr*-4S3/3′ enhancer from seven different *Drosophila* species revealed three highly conserved boxes (I to III). Identified and verified binding sites for all eight co-regulatory transcription factors are highlighted in different colours.
(3.37 MB TIF)

Figure S4 Identification and verification of binding sites for co-regulatory transcription factors in the *rpr*-4S3/3′ enhancer. (A) EMSA for mapping of Slp1 binding site in the *rpr*-4S3/3′ enhancer using box 3 (as shown in Figure S3) as shift probe. EMSA was performed using no protein (P), translation lysate only (L) and lysate with Slp1 protein (S). c_30_ to c_36_ represent competitor oligonucleotides with consecutive base-pairs mutated. Competition experiments revealed that sequences mutated in the oligonucleotides c_33_ include binding site for the Slp1 protein. The purple arrowheads indicate specific DNA-protein complexes containing Slp1 protein. The asterisk indicates a complex with lysate protein seen also in the control. (A′) EMSA using box 3 (as shown in Figure S3) and no protein (P), translation lysate (L), lysate with Slp1 protein (S) and lysate with Dfd protein (D). To test specificity of binding of Slp1 protein to the DNA fragment, competitor oligonucleotides for the mapped Slp1 binding site were used either in their wild-type (c_wt_) or mutant (c_mt_) sequence versions. The purple arrowheads indicate specific DNA-protein complexes containing Slp1 protein. Note that in the competitor oligonucleotides only the binding site sequence for the Slp1 protein is mutated, but not for the Dfd binding site sequence. (B) EMSA for mapping of Disco binding site 1 in the *rpr*-4S3/3′ enhancer using box 1 (as shown in Figure S3) as shift probe. EMSA was performed using no protein (P), translation lysate only (L) and lysate with Disco protein (C). c_1_ to c_7_ represent competitor oligonucleotides with consecutive base-pairs mutated. Competition experiments revealed that sequences mutated in the oligonucleotides c_3_ and c_4_ include binding site for the Disco protein. The turquoise arrowheads indicate the DNA-protein complexes containing Disco protein. The asterisk indicates a complex with lysate protein seen also in the control. (B′) EMSA using box 1 (as shown in Figure S3) and no protein (P), translation lysate (L), lysate with Disco protein (C) and lysate with Dfd protein (D). To test specificity of binding of Disco protein to the DNA fragment, competitor oligonucleotides for the mapped Disco binding site were used either in their wild-type (c_wt_) or mutant (c_mt_) sequence versions. The red and turquoise arrowheads indicate the specific DNA-protein complexes containing either Dfd or Disco protein, respectively. The asterisk indicates a complex with lysate protein seen also in the control. Note that in the competitor oligonucleotides only the binding site sequence for the Disco protein is mutated, but not for the Dfd binding site sequence. (C) EMSA for mapping of Brk binding site in the *rpr*-4S3/3′ enhancer using box 2 (as shown in Figure S3) as shift probe. EMSA was performed using no protein (P), translation lysate only (L) and lysate with Brk protein (B). c_8_ to c_13_ represent competitor oligonucleotides with consecutive base-pairs mutated. Competition experiments revealed that sequences mutated in the oligonucleotide c_10_ and c_11_ include binding site for the Brk protein. The blue arrowheads indicate specific DNA-protein complexes containing Brk protein. The asterisks indicate complexes with lysate protein seen also in the control. (C′) EMSA using box 2 (as shown in Figure S3) and no protein (P), translation lysate (L), lysate with Brk protein (B) and lysate with Dfd protein (D). To test specificity of binding of Brk protein to the DNA fragment, competitor oligonucleotides for the mapped Brk binding site were used either in their wild-type (c_wt_) or mutant (c_mt_) sequence versions. The red and blue arrowheads indicate the specific DNA-protein complexes containing either Dfd or Brk protein, respectively. The asterisks indicate complexes with lysate protein seen also in the control. Note that in the competitor oligonucleotides only the binding site sequence for the Brk protein is mutated, but not for the Dfd binding site sequence. (D) EMSA using box 2 (as shown in Figure S3) and no protein (P), translation lysate (L), lysate with En protein (N) and lysate with Dfd protein (D). To test specificity of binding of En protein to the DNA fragment, competitor oligonucleotides for En binding site were used either in their wild-type (c_wt_) or mutant (c_mt_) sequence versions. The red and light green arrowheads indicate the specific DNA-protein complexes containing either Dfd or En protein, respectively. Note that in the competitor oligonucleotides only the binding site sequence for the En protein is mutated, but not for the Dfd binding site sequence. (E) EMSA using box 2 (as shown in Figure S3) and no protein (P), translation lysate (L), lysate with Apt protein (A) and lysate with Dfd protein (D). To test specificity of binding of Apt protein to the DNA fragment, competitor oligonucleotides for Apt binding site were used either in their wild-type (c_wt_) or mutant (c_mt_) sequence versions. The red and light purple arrowheads indicate the specific DNA-protein complexes containing either Dfd or Apt protein, respectively. Note that in the competitor oligonucleotides only the binding site sequence for the Apt protein is mutated, but not for the Dfd binding site sequence.
(3.72 MB TIF)
