# Correction: Multifactorial Regulation of a Hox Target Gene

**DOI:** 10.1371/annotation/f89de76b-0e06-4086-994c-20d64c238a46

**Published:** 2009-03-20

**Authors:** Petra Stöbe, M. A. Sokrates Stein, Anette Habring-Müller, Daniela Bezdan, Aurelia L. Fuchs, Stefanie D. Hueber, Haijia Wu, Ingrid Lohmann

The second author's name was incorrectly listed. The correct name is: M. A. Sokrates Stein. The correct citation is: Stöbe P, Stein MAS, Habring-Müller A, Bezdan D, Fuchs AL, et al. (2009) Multifactorial Regulation of a Hox Target Gene. PLoS Genet 5(3): e1000412. doi:10.1371/journal.pgen.1000412

