# Supplementary figures and images for: Multifactorial Regulation of a Hox Target Gene
Source: PLoS Genet. 2009 Mar 13;5(3):e1000412. doi: 10.1371/journal.pgen.1000412 (PMC2646128; doi:10.1371/journal.pgen.1000412)

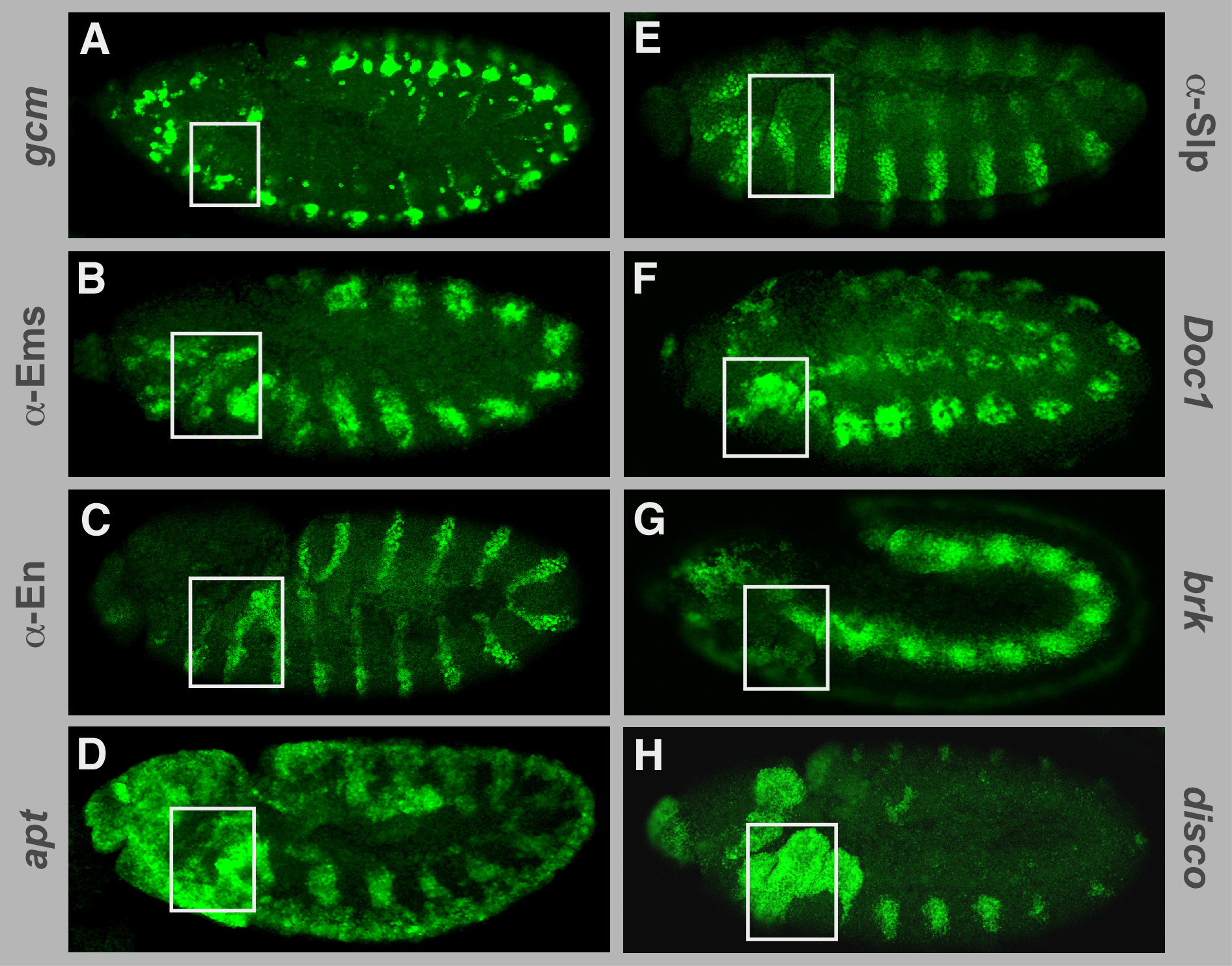

Supplement: Figure S1 — Expression patterns of the identified transcription factors in stage 11 wild-type embryos. For the following genes, antibody stainings are shown: ems (B), en (C) and slp1 (E). Due to the unavailability or inactivity of antibodies, in situ hybridizations for the following genes are shown: gcm (A), apt (D), Doc1 (F), brk (G) and disco (H). Boxes in (A to H) highlight the maxillary segment. (2.53 MB TIF) [file pgen.1000412.s001.tif]

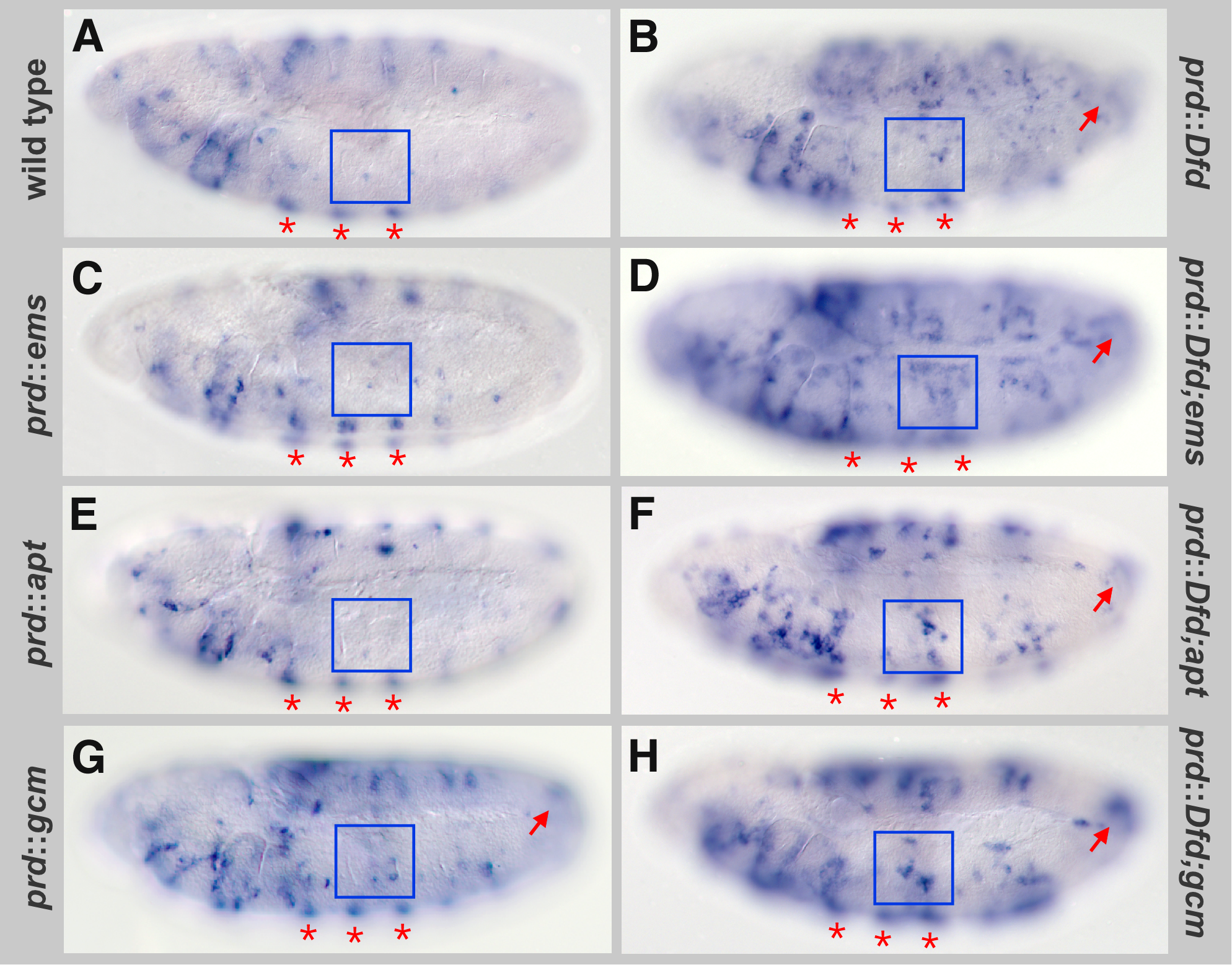

Supplement: Figure S2 — Identified transcription factors modulate rpr expression when mis-expressed. rpr RNA in situ hybridizations in stage 11 embryos with the following genotypes are shown: (A) wild type, (B) prd::Dfd, (C) prd::ems, (D) prd::Dfd;ems, (E) prd::apt, (F) prd::Dfd;apt, (G) prd::gcm, (H), prd::Dfd;gcm. Co-expression of Ems, Apt and Gcm with Dfd enhances Dfd-dependent ectopic rpr induction (D, F and H), whereas Gcm is able to ectopically induce rpr expression alone. To select identical stages, three characteristic spots of rpr expression in the thoracic segments normally seen in stage 11 wild-type embryos (marked by three asterisks) were used. In (B to H) one spot of ectopic rpr expression at the very posterior end in the prd-GAL4 over-expression embryos is marked by a red arrow, the blue box outlines an additional stripe of rpr RNA expression in the T3 primordium. (6.46 MB TIF) [file pgen.1000412.s002.tif]

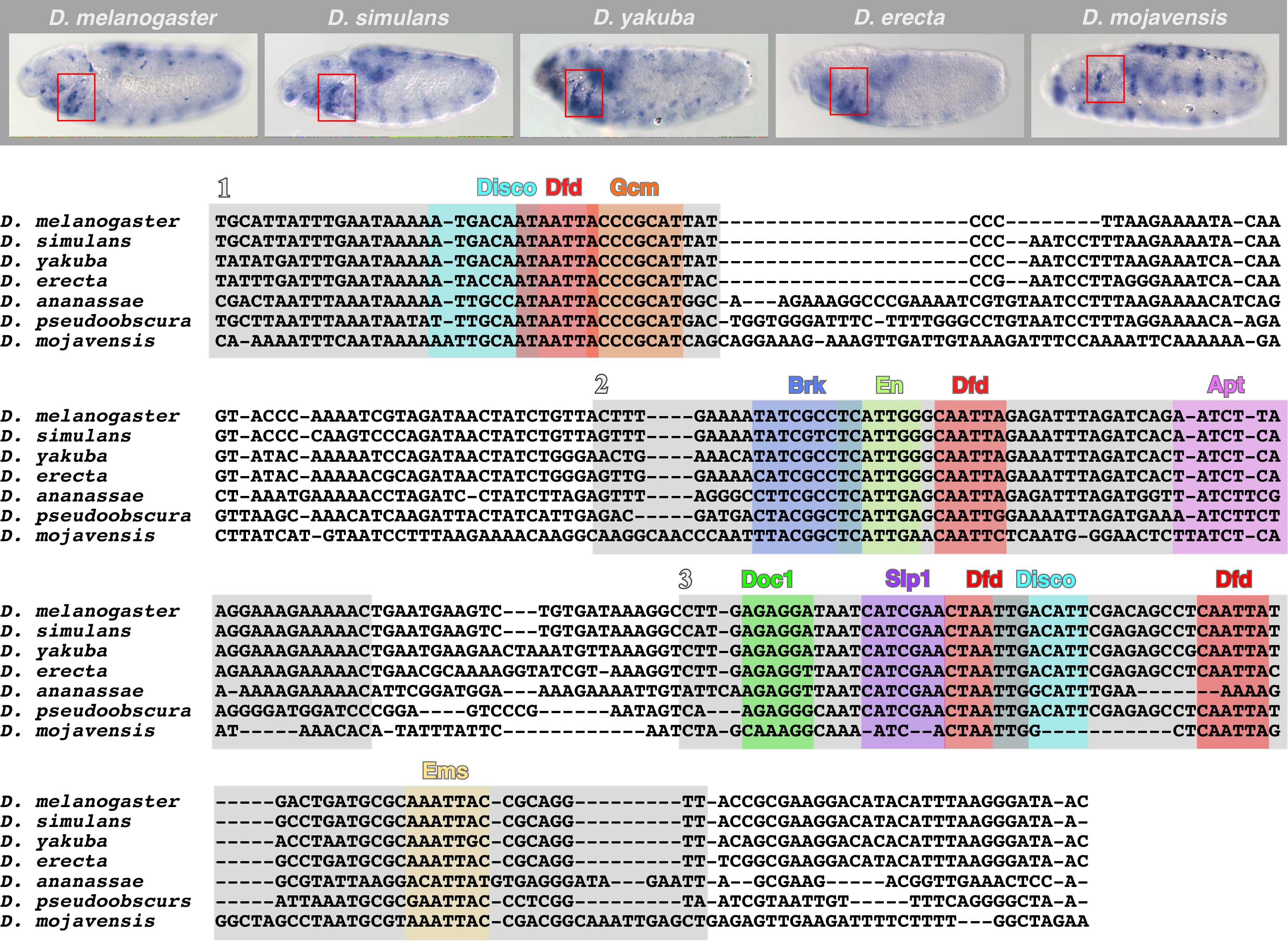

Supplement: Figure S3 — Identification of conserved regulatory elements in the rpr-4S3/3′ enhancer by phylogenetic footprint analysis. Upper: rpr RNA expression in stage 11 embryos of different Drosophila species used for the phylogenetic footprint analysis. In situ hybridization experiments with species-specific probes show that rpr is expressed in the anterior part of all five Drosophila species (D. melanogaster, D. simulans, D. yakuba, D. erecta, D. mojavensis). The red boxes highlight the maxillary segment. Bottom: Alignment of the rpr-4S3/3′ enhancer from seven different Drosophila species revealed three highly conserved boxes (I to III). Identified and verified binding sites for all eight co-regulatory transcription factors are highlighted in different colours. (3.37 MB TIF) [file pgen.1000412.s003.tif]

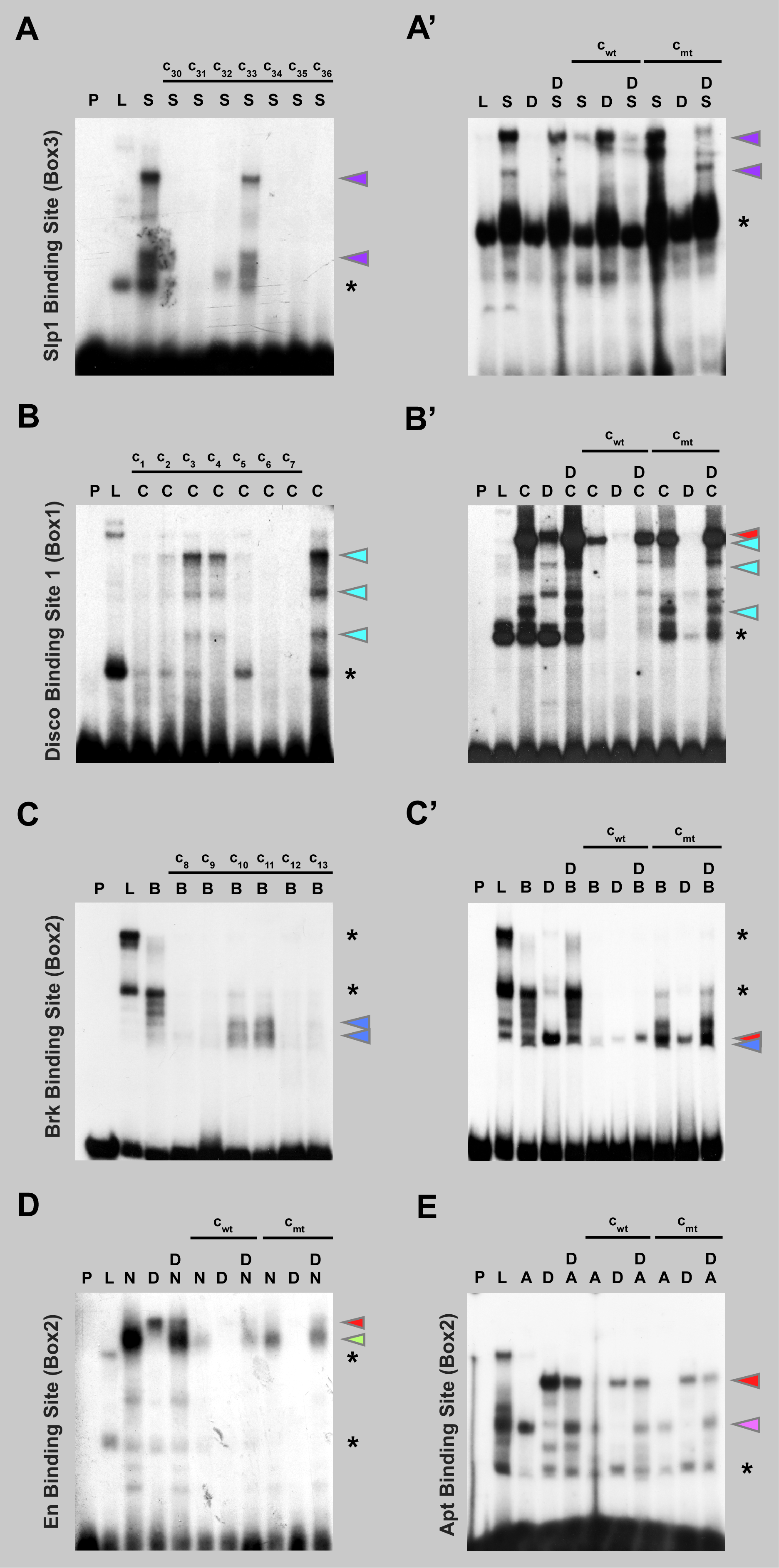

Supplement: Figure S4 — Identification and verification of binding sites for co-regulatory transcription factors in the rpr-4S3/3′ enhancer. (A) EMSA for mapping of Slp1 binding site in the rpr-4S3/3′ enhancer using box 3 (as shown in Figure S3) as shift probe. EMSA was performed using no protein (P), translation lysate only (L) and lysate with Slp1 protein (S). c30 to c36 represent competitor oligonucleotides with consecutive base-pairs mutated. Competition experiments revealed that sequences mutated in the oligonucleotides c33 include binding site for the Slp1 protein. The purple arrowheads indicate specific DNA-protein complexes containing Slp1 protein. The asterisk indicates a complex with lysate protein seen also in the control. (A′) EMSA using box 3 (as shown in Figure S3) and no protein (P), translation lysate (L), lysate with Slp1 protein (S) and lysate with Dfd protein (D). To test specificity of binding of Slp1 protein to the DNA fragment, competitor oligonucleotides for the mapped Slp1 binding site were used either in their wild-type (cwt) or mutant (cmt) sequence versions. The purple arrowheads indicate specific DNA-protein complexes containing Slp1 protein. Note that in the competitor oligonucleotides only the binding site sequence for the Slp1 protein is mutated, but not for the Dfd binding site sequence. (B) EMSA for mapping of Disco binding site 1 in the rpr-4S3/3′ enhancer using box 1 (as shown in Figure S3) as shift probe. EMSA was performed using no protein (P), translation lysate only (L) and lysate with Disco protein (C). c1 to c7 represent competitor oligonucleotides with consecutive base-pairs mutated. Competition experiments revealed that sequences mutated in the oligonucleotides c3 and c4 include binding site for the Disco protein. The turquoise arrowheads indicate the DNA-protein complexes containing Disco protein. The asterisk indicates a complex with lysate protein seen also in the control. (B′) EMSA using box 1 (as shown in Figure S3) and no protein (P), transl [file pgen.1000412.s004.tif]
